# Use of over-the-scope clip for ulcer hemostasis after failure of both surgery and interventional therapy: A case report

**DOI:** 10.1097/MD.0000000000022646

**Published:** 2020-10-09

**Authors:** Yong-Kang Lai, Chun-Yan Peng, Gui-Hai Guo, Xu Shu

**Affiliations:** Department of Gastroenterology, The First Affiliated Hospital of Nanchang University, Nanchang, Jiangxi, China.

**Keywords:** over-the-scope clip, radiological interventions, surgery, ulcer bleeding, upper gastrointestinal bleeding

## Abstract

**Rationale:**

Non-variceal gastrointestinal bleeding is a common critical disease worldwide, and according to relevant guidelines, surgery and interventional treatment are the final therapies. However, few studies have reported on therapeutic strategies to employ when the ultimate treatment fails. This report offers a reasonable option for hemostasis after surgery and interventional treatment both fail.

**Patient concerns:**

A 47-year-old man with recurrent bleeding had undergone endoscopy, surgery, and interventional therapy; however, effective hemostasis was not achieved.

**Diagnosis:**

This patient's clinical manifestations and typical gastroscopic findings confirmed duodenal bulb ulcer with hemorrhage

**Interventions:**

A Billroth II + Bancroft operation, interventional treatment, and endoscopic hemostasis with an over-the-scope clip (OTSC) system were administered.

**Outcomes:**

The bleeding was successfully controlled, and the patient remained well during long-term follow-up.

**Lessons:**

The OTSC system can represent a reasonable option for ulcer hemostasis after surgery when other interventional therapies have failed.

## 1 Introduction

Upper gastrointestinal bleeding is a significant cause of hospitalization and mortality worldwide,^[1]^ and despite advances in medications and therapeutic techniques, the rates of rebleeding and mortality remain high.^[2]^ Endoscopic hemostasis is the first choice of treatment for patients with upper gastrointestinal bleeding,^[3]^ and surgical and radiological interventions are secondary measures.^[4,5]^ However, surgical and radiological interventions sometimes fail to stop the bleeding. In such cases, it is unclear what the next step should be, such as a second endoscopic hemostasis procedure, additional interventional therapies, or an additional surgery. Research on this topic has been scarce.

The over-the-scope clip (OTSC) system (Ovesco Endoscopy AG, Tübingen, Germany) is a novel endoscopic device that can be used to manage leaks and fistulas,^[6,7]^ remove submucosal tumors,^[8]^ and close perforations.^[9]^ In addition, the efficacy of this system in treating high-risk gastrointestinal bleeding has been widely reported.^[10,11]^ We used the OTSC system to successfully achieve hemostasis in a patient who was still bleeding from a lesion after surgical and interventional treatments. We then performed long-term follow-up observations.

## 2 Case report

**Figure 1:**
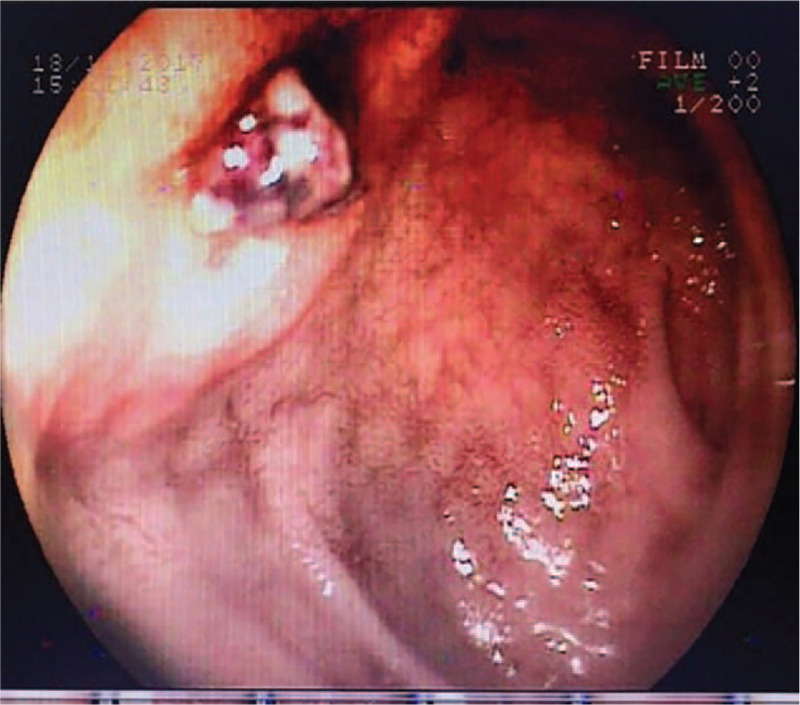
Endoscopy performed at a local hospital. Endoscopic images suggested that an ulcer (0.8 cm × 0.8 cm) was on the anterior wall of the duodenal bulb.

A 47-year-old man had intermittent dull pain in the upper abdomen after eating greasy food for 2 days associated with melena twice a day before he was admitted to the local hospital. During hospitalization, the patient experienced hemorrhagic shock and underwent an initial emergency gastroscopy. An ulcer (0.8 cm × 0.8 cm) was identified on the anterior wall of the duodenal bulb (Fig. 1). The ulcer had a large area, and its anatomical location was difficult to approach. Moreover, the ulcer caused arterial bleeding, thus traditional endoscopic hemostasis was difficult to achieve; thus, effective hemostasis was not attained. Due to the large amount of bleeding, poor endoscopic field of vision, and small operating space, the ulcer could not be treated. Thus, the patient was immediately transferred to surgery, and a gastroenterostomy (Billroth II) + Bancroft operation was performed. During the operation, ulcers with scabs were observed near the descending part of the duodenal bulb and the ulcerated area was deep. After determining the area for surgical excision, it was still difficult to fully expose the intestinal tubes of the duodenal ulcer from the outside; therefore, the surgeon at the local hospital selected the Billroth II + Bancroft operation. The bleeding stopped after the operation.

**Figure 2:**
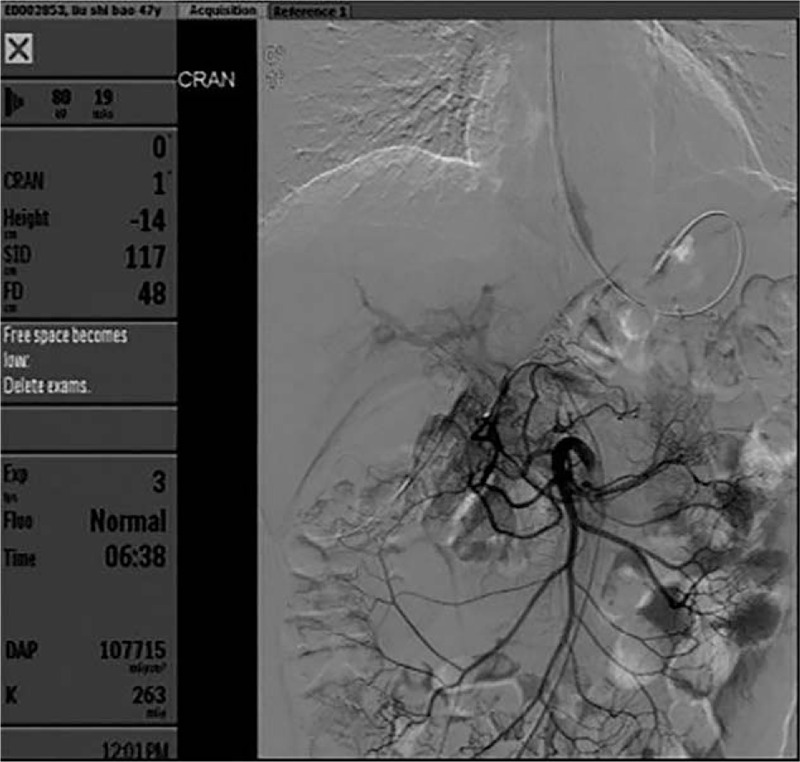
Digital subtraction angiography (DSA) performed at the First Affiliated Hospital of Nanchang University for treatment. Bleeding was not found in the gastroduodenal artery or superior mesenteric artery.

**Figure 3:**
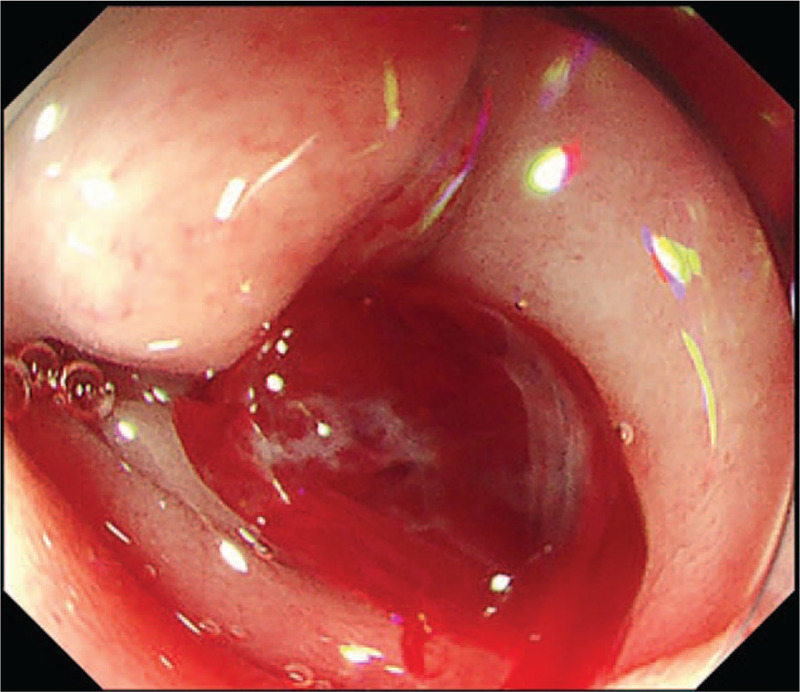
JI enteroscope (PCF-Q260JI, Olympus, Tokyo, Japan) was used innovatively. Fortunately, blood clots were observed in the input loop and residual blood vessels were observed near the blind end. Then, the OTSC system was used to treat the lesion. Repeated flushing showed no bleeding. OTSC = over-the-scope-clip.

**Figure 4:**
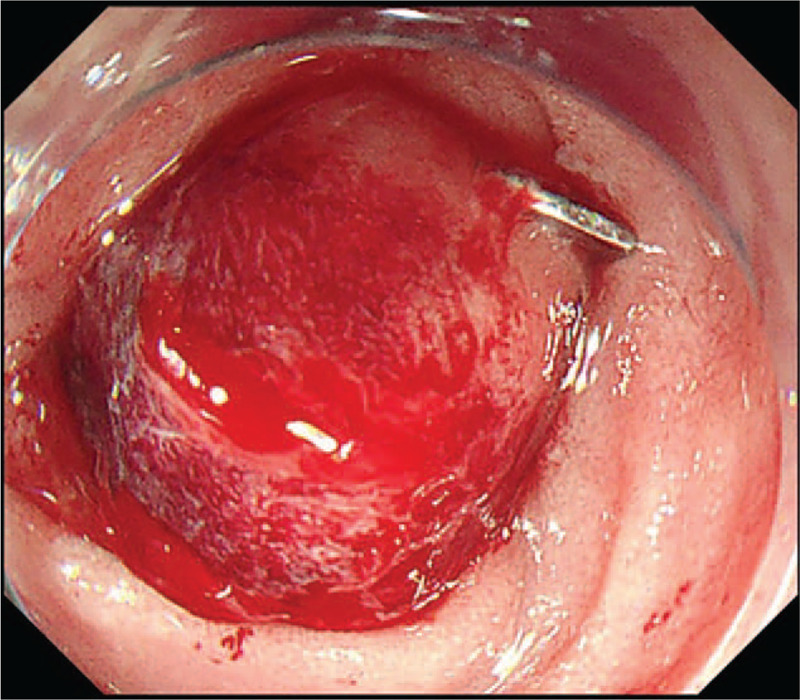
OTSC system used to treat the lesion; repeated flushing showed no bleeding. OTSC = over-the-scope-clip.

**Figure 5:**
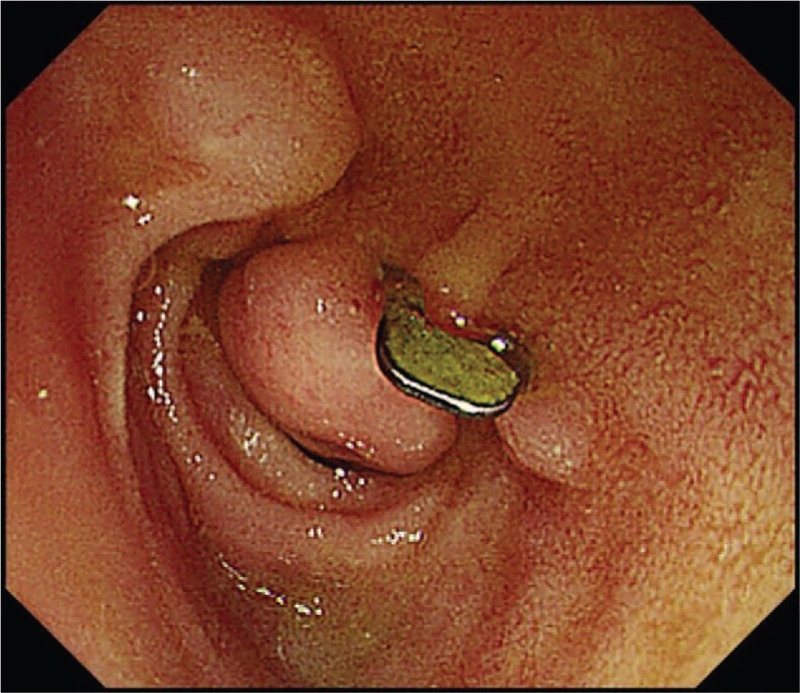
One-year follow-up endoscopy showed that the patient did not bleed again.

Seven days postoperatively, the patient again experienced melena, which suggested that the ulcer had begun bleeding again. He was immediately transferred to the Department of Gastroenterology at the First Affiliated Hospital of Nanchang University, where he underwent endoscopic hemostasis for the second time. However, no lesion was observed. Digital subtraction angiography (DSA) was also performed, and it revealed no signs of hemorrhage (Fig. 2). The patient's condition was stable after blood transfusion and acid inhibition therapy. Five days after he was admitted to our hospital, the patient suddenly vomited a large quantity of blood (approximately 800 mL), and his hemoglobin level dropped to 57 g/L. An emergency endoscopy was performed, and a large amount of fresh blood was observed in the esophagus and residual stomach. Repeated washing of the anastomotic site showed that there were no bleeding points. Then, we used a JI enteroscope (PCF-Q260JI, Olympus, Tokyo, Japan), which can extend deeper than a normal gastroscope. Fortunately, blood clots were observed in the input loop, and residual blood vessels were observed near the blind end (Fig. 3). Next, the positioning for the OTSC system was confirmed above the lesion. By rotating the wheel, which was attached to the endoscope, the OTSC was quickly deployed to the target site and the hemostasis process was completed. Repeated flushing revealed no additional blood (Fig. 4), indicating that the bleeding had stopped. We performed follow-up examinations at 5 months, 10 months, and 1 year after endoscopy (Fig. 5). Rebleeding was not observed in any of these examinations.

## 3 Discussion

We report on a patient treated with the OTSC system and find that the system successfully stopped rebleeding after the failure of both surgical and interventional treatments. In this patient, the ulcer's anatomical location was difficult to approach, and the large amount of bleeding, poor endoscopic field of vision, and small operating space made traditional endoscopic hemostasis difficult to fully achieve. In addition, the patient was in worse condition because of the ulcer's bleeding. The first emergency endoscopy failed to stop the bleeding; consequently, surgery was immediately performed to achieve hemostasis. To avoid the complications of a Billroth II-type gastric resection,^[12]^ the local hospital chose a Bancroft operation to address the duodenal ulcer. Although the Billroth II operation is the cornerstone for the management of patients with uncontrolled bleeding,^[13]^ the ulcer was not completely resected. When the patient underwent an emergency endoscopy for the second time at our hospital, the ulcer was located so deeply that the exact location could not be identified using the normal endoscopy procedure. Therefore, DSA was used to look for the source of the gastrointestinal bleeding; however, we were still unable to identify the ulcer. The reasons for this failure might be as follows: the bleeding arteries were too small to be observed; the lesion bled intermittently and had temporarily stopped bleeding when we performed the DSA procedure; and DSA might not be an effective technique for identifying bleeding areas. Therefore, we made the innovative choice to use the JI enteroscope and finally found the lesion near the blind side when the patient again experienced hematemesis. Then, the OTSC system was used to successfully stop the bleeding.

The OTSC system is a novel and full-thickness suturing device that can close lesions, such as perforations,^[14]^ fistulas,^[6]^ and sites of gastrointestinal bleeding.^[15]^ Its characteristics, including a high-elasticity nickel alloy, shape-memory function, and strong and persistent organizational grip, render the OTSC system effective for the continuous closure of wounds, which is why the OTSC system was able to stop the patient's ulcer from bleeding after the failure of both surgery and DSA.

To date, several studies have reported on the use of the OTSC system as a rescue treatment for refractory bleeding after endoscopic hemostasis.^[10,16–18]^ However, in those studies, surgery was considered the final choice, and whether endoscopic hemostasis could be attempted again after the failure of surgery and interventional therapy was not indicated. Reports of rescue therapy after the failure of surgical and interventional treatments have been rare. This study is innovative in the use of J-type enteroscopy to detect a deep ulcer and the OTSC system to close the wound after the failure of surgical treatment, which could thus serve as an alternative treatment for hemostasis after the failure of surgery and interventional therapy.

In conclusion, the application of the OTSC system is a reasonable option for controlling ulcer rebleeding after the failure of both surgery and interventional therapy, especially for ulcers with deep anatomical locations, large amounts of bleeding, and small bleeding arteries. This is the first report suggesting that surgical and interventional therapies are not the last line of defense against ulcer bleeding. Thus, for cases of ulcers in which both surgical and interventional therapies fail, the OTSC system may represent a treatment option.

## Author contributions

Xu Shu designed the report, approved the final submission, and clinically managed the patient; Yong-Kang Lai collected the data, analyzed the relevant information, and wrote the manuscript; and Chun-Yan Peng and Gui-Hai Guo clinically managed the patient.

## Abbreviations

Abbreviations: DSA = digital subtraction angiography, GI = gastrointestinal, OTSC = over-the-scope-clip.
